# Performance of non-invasive prenatal screening for 15q11q13 copy number variations in a cohort of 90,693 pregnancies

**DOI:** 10.3389/fgene.2026.1937108

**Published:** 2026-10-09

**Authors:** Diyu Chen, Liang Hu, Hongyan Niu, Zhenming Li, Shuxian Zeng, Xiaoyi Cong, Pingnan Sun, Weiqiang Liu

**Affiliations:** 1 Central Laboratory (Genetics Lab), Longgang District Maternity and Child Healthcare Hospital of Shenzhen City (Affiliated Shenzhen Women and Children’s Hospital (Longgang) of Shantou University Medical College), Shenzhen, China; 2 Longgang District Key Laboratory for Birth Defects Prevention, Shenzhen, China; 3 GuangDong Engineering Technology Research Center of Genomics and Precision Imaging, Shenzhen, China; 4 Stem Cell Research Center, Shantou University Medical College, Shantou, China

**Keywords:** 15q11q13 duplication syndrome, angelman syndrome, copy number variations (CNVs), non-invasive prenatal screening (NIPS), Prader-Willi syndrome

## Abstract

**Background:**

Deletions and duplications within the 15q11q13 chromosomal region are associated with Prader-Willi syndrome, Angelman syndrome, and 15q11q13 duplication syndrome. However, prenatal ultrasound findings were unremarkable in the majority of affected fetuses. The aim of this study was to evaluate the efficacy of non-invasive prenatal screening (NIPS) in identifying these syndromes in 90,693 pregnancies within the general population.

**Methods:**

We retrospectively analyzed 90,693 pregnant women who underwent NIPS at Longgang Maternal and Child Health Hospital in Shenzhen from November 2022 to January 2026. For high-risk 15q11q13 copy number variations (CNVs) identified, we performed chromosomal microarray analysis (CMA) on amniotic fluid, followed by methylation-specific multiplex ligation-dependent probe amplification (MS-MLPA) or further CMA to determine the CNVs’ genetic origin, with subsequent follow-up.

**Results:**

NIPS screening identified 4 high-risk cases for 15q11q13 deletion and 7 for duplication. Among the deletion cases, 3 were confirmed by CMA to have a 5–6 Mb deletion (2 maternal, 1 unverified), while 1 declined further testing. For duplication, 6 underwent amniocentesis, with CMA confirming 5 cases (2 maternal, 2 paternal, 1 unverified), 1 normal result, and 1 declined further testing. Of the 8 true-positive cases, 7 had normal ultrasounds, but 1 fetus with duplication exhibited growth retardation. Pregnancy outcomes included 6 terminations, one delivery with paternal duplication showing mild language delay at nearly 2 years, and another with maternal duplication lost to follow-up.

**Conclusion:**

NIPS can effectively detect CNVs at the 15q11q13 locus with a high positive predictive value, but its sensitivity, specificity, negative predictive value, and false-negative rate could not be determined in this study due to the lack of confirmatory testing in low-risk cases. Invasive prenatal diagnosis remains essential for validation.

## Introduction

The 15q11q13 chromosomal region is characterized by the presence of low-copy repeats, making it a hotspot for chromosomal rearrangements and susceptible to copy number variations (CNVs). This region encompasses five breakpoints (BP1-BP5), with the typical ∼5–6 Mb deletion occurring between the BP1 and BP3 intervals. This deletion is the primary genetic cause of Angelman syndrome (AS) and Prader-Willi syndrome (PWS), with the resulting phenotype contingent upon the parental origin of the deleted segment. Approximately 70% of PWS and AS cases are attributed to deletions of the paternal or maternal 15q11q13 region, respectively (Butler, 2026). AS is primarily characterized by severe intellectual disability, motor and language impairments, epilepsy, and an unusually happy demeanor (Rotaru et al., 2020). PWS is marked by neonatal hypotonia and feeding difficulties in early infancy, followed by hyperphagia and obesity in childhood (Mahmoud et al., 2023). Duplications within the 15q11q13 region are linked to 15q11q13 duplication syndrome (Dup15q syndrome), which is primarily associated with autism spectrum disorder, intellectual disability, developmental delay, and language impairment (Bisba et al., 2024).

Early diagnosis is critically important for the prevention and management of AS, PWS, and Dup15q syndrome. However, the majority of fetuses affected by AS, PWS, and Dup15q syndrome typically exhibit no abnormal findings during prenatal ultrasound examinations, which poses a significant challenge to early detection. Therefore, there is a pressing need to develop effective screening methods for detecting CNVs in the 15q11q13 region. Recent advancements in sequencing depth and algorithmic optimization have improved the performance of non-invasive prenatal screening (NIPS). While NIPS has demonstrated high sensitivity and specificity for trisomy 21, trisomy 18, and trisomy 13 (Alberry et al., 2021), it also shows promise for detecting CNVs (Bedei et al., 2021). Previous studies have shown that NIPS can achieve a screening efficiency of up to 90.9% for chromosomal deletions or duplications with fragment sizes of 5 Mb or larger (Li et al., 2016). To date, there have been limited studies investigating CNVs in the 15q11q13 region using NIPS.

In this study, NIPS was conducted on cell-free fetal DNA (cffDNA) obtained from the peripheral blood of 90,693 pregnant women. The screening identified 4 high-risk cases of 15q11q13 deletion and 7 high-risk cases of 15q11q13 duplication, indicating an elevated risk of AS, PWS, and Dup15q syndrome in the fetuses. Subsequent prenatal diagnostic results confirmed the utility of NIPS for detecting CNVs in this genomic region, demonstrating a favorable positive predictive value (PPV).

## Materials and methods

### Subjects

From November 2022 to January 2026, a total of 90,693 pregnant women who underwent NIPS at Shenzhen Longgang District Maternal and Child Health Hospital were enrolled in this study. This research received approval from the Medical Ethics Committee of Shenzhen Longgang District Maternal and Child Health Hospital (No. KYXM-2023–045). Written informed consent was obtained from all participants prior to the testing.

### CffDNA extraction, library enrichment, and sequencing

CffDNA was extracted from 5 mL peripheral blood samples using a DNA extraction kit provided by BGI, Wuhan, China. The samples underwent centrifugation at 16,000 g for 10 min at 4 °C. To enrich larger cffDNA fragments, 32 µL of the cffDNA solution was mixed with 39 µL of AMPureXP magnetic beads (Beckman Coulter, United States). An additional 30 µL of AMPureXP magnetic beads was added to capture smaller cffDNA fragments. Following two washes with 75% ethanol, 42 µL of elution buffer was used to elute the DNA. For sequencing, the DNA libraries were denatured into single strands, followed by rolling amplification using DNA nanoballs polymerase. The DNA nanoballs were subsequently loaded onto a chip, which was incubated and then sequenced. Sequencing was conducted using the MGIseq2000 system.

For quality control, the average sequencing depth was determined based on the number of unique reads; in our workflow, the minimum unique reads for each sample were more than 5 million. The fetal fraction threshold was set at 3.5%, as fetal fraction below this level has been associated with increased failure rates and reduced accuracy. The Z-score threshold for calling aneuploidy was >3 for trisomies 21, 18, and 13, and sex chromosome aneuploidies; borderline Z-scores triggered re-construction or re-sampling to reduce false positives. For CNV detection, the minimum resolvable size was 1 Mb at the genome-wide level. These parameters were implemented to ensure the robustness and accuracy of the NIPS assay.

### Bioinformatic analysis

Bioinformatic analysis was performed using Halos software with GRCh37 as the reference genome, and a z-score threshold of 3 was applied to identify trisomy 13, 18, and 21. The Hidden Markov Model (R package HMM, v1.0.1) was employed to detect fetal CNVs.

### Algorithms for CNV analysis

The human reference genome (hg19) was divided into 50-bp windows for read alignment. Sequencing reads were mapped to hg19, and the corrected read number (CRN) was determined for each window. To standardize read counts, a Z-score was calculated for each window: *Z*_*win*_ *= (CRN*_*win*_ *− CRN*_*mean*_ *)/SD*_*CRN*_, where CRN_win_ is the window’s CRN, CRN_mean_ is the average CRN across samples, and SD_CRN_ is the standard deviation of the CRN.

### Karyotyping analysis

Amniotic fluid cells obtained via amniocentesis were aseptically inoculated into T-25 culture flasks and maintained at 37 °C within a 5% CO_2_ incubator. Following a culture period of 6–8 days, depending on the cell growth status, chromosome slides were prepared using standard protocols. These slides were subsequently examined on a Leica automated karyotype scanning platform, and metaphases with well-defined banding patterns were selected for karyotypic analysis.

### Chromosomal microarray analysis (CMA)

DNA extraction was conducted using the QIAamp DNA Extraction Kit (Qiagen, Germany). The isolated DNA was subsequently analyzed using the CytoScan 750K Array (Affymetrix, United States), which is a SNP-based microarray platform. Following the manufacturer’s standard protocol, the DNA samples underwent enzymatic digestion, ligation, PCR amplification, purification, fragmentation, signal labeling, hybridization, and subsequent washing, staining, and scanning, culminating in data analysis. The data analysis was executed using the ChAS 4.3 software. For comparative analysis, the resultant data were cross-referenced against several public databases, including UCSC Genome Browser, DECIPHER, Database of Genomic Variants, and ClinGen.

### Methylation-specific multiplex ligation-dependent probe amplification (MS-MLPA) analysis

MS-MLPA was performed with the SALSA MLPA Probemix ME028 kit (MRC-Holland) according to the supplier’s instructions. The probes target the 15q11 region for methylation and copy number assessment. Data were analyzed using Coffalyser.Net software with the appropriate lot-specific sheet.

### Ultrasound examinations

The ultrasound examination was conducted using the Voluson E10 system (GE Medical Systems), operating at a scanning frequency range of 1–5 MHz.

### Pregnancy outcome follow-up

A postnatal follow-up was conducted for cases with NIPS results suggesting deletions or duplications within the 15q11q13 chromosomal region. This monitoring was conducted for up to a period of 2 years postpartum.

## Results

### Analysis of NIPS results for 15q11q13 CNVs

Between November 2022 and January 2026, a cohort of 90,693 pregnant women underwent NIPS at our hospital. Within this cohort, 4 cases were identified as high-risk for a 15q11q13 deletion, and 7 cases were identified as high-risk for a duplication within the same chromosomal region (Figure 1). NIPS screening revealed 1 case of a deletion fragment at 15q11q13 ≥ 5 Mb and 2 cases of deletion fragments between 4 Mb and 5 Mb. For duplications, 6 cases had fragments ≥5 Mb, and 1 case had a fragment between 3 Mb and 4 Mb (Table 1).

**FIGURE 1:**
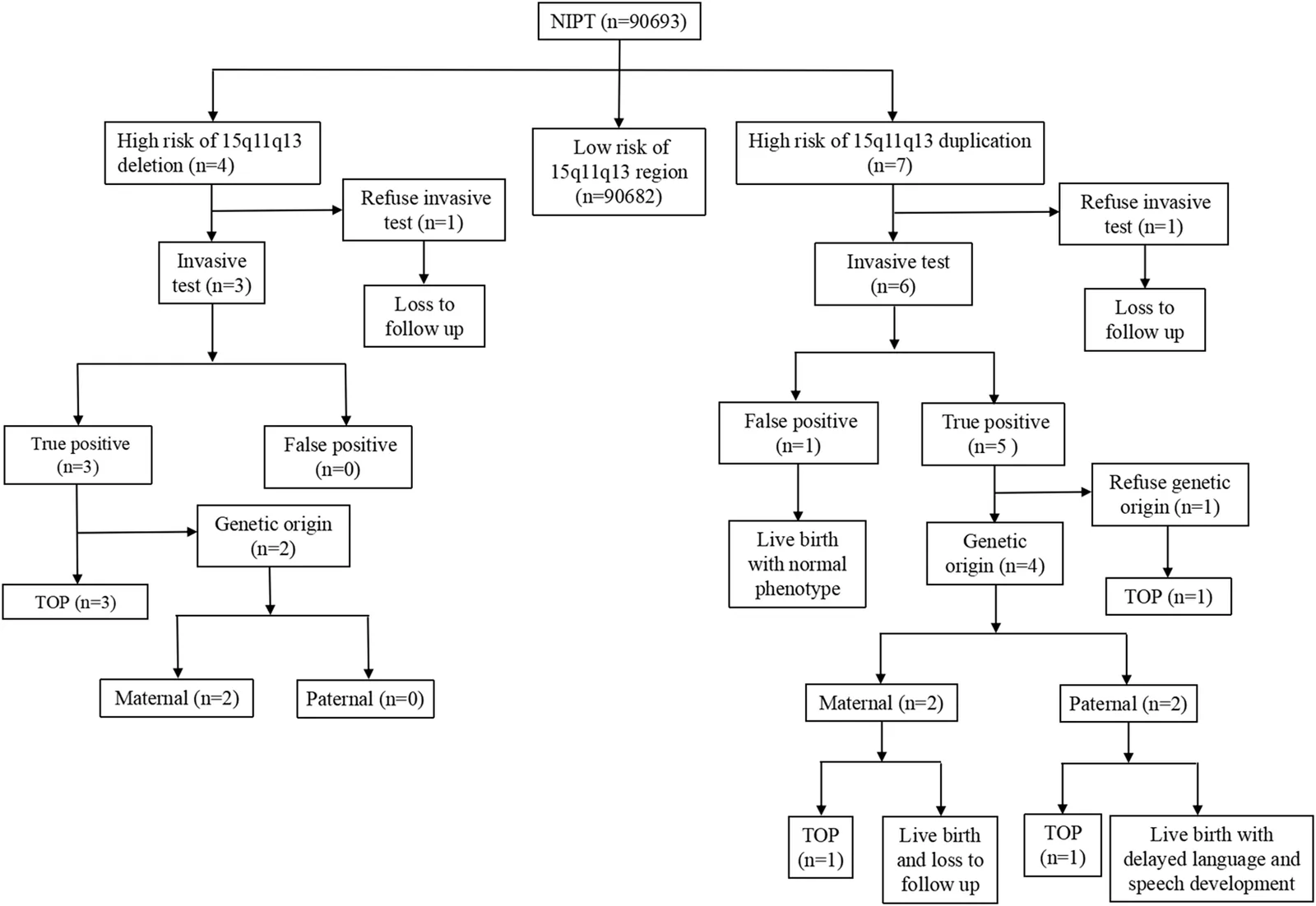
Flowchart of Routine NIPS for 15q11q13 CNVs in 90,693 Pregnant Women. This figure illustrates the methodology and outcomes of non-invasive prenatal screening (NIPS) for 15q11q13 copy number variants (CNVs) in a cohort of 90,693 pregnant women from the general population. It provides detailed information on the screening results, Chromosomal Microarray Analysis (CMA) confirmation, and subsequent clinical management.

**TABLE 1:** Detailed information of 11 cases with NIPS 15q11q13 region high-risk screening.

Case no.	Age	Gestational age (week)	cffDNA (%)	NIPS results	Ultrasound findings	Karyotyping	CMA results	CNV size	Genetic origin	Diagnosis	Pregnancy outcome
1	32	16^+5^	16.093	del (15q11.2-q13.1,5.12M)	Normal	Normal	arr [GRCh37] 15q11.2q13.1 (23,290,788_28,540,345)x1	5250 Kb	Maternal	Angelman syndrome	Terminate of pregnancy
2	28	13	16.209	del (15q11.2-q13.1,4.78M)	Normal	Normal	arr [GRCh37] 15q11.2q13.1 (23,290,788_28,527,734)x1	5237 Kb	Maternal	Angelman syndrome	Terminate of pregnancy
3	35	13^+5^	23.1	del (15q11.2-q13.1,5.43M)	Normal	Normal	arr [GRCh37] 15q11.2q13.1 (23,290,788_28,545,355)x1	5255 Kb	Unknown	Angelman syndrome or prader-willi syndrome	Terminate of pregnancy
4	31	13^+2^	28.43	del (15q11.2-q13.1,4.06M)	Normal	**—**	**—**	**—**	**—**	**—**	Loss to follow up
5	26	15^+5^	19.303	dup (15q11.2-q13.1,5.12M)	Normal	46,XN,dup (15) (q11.2q13)	arr [GRCh37] 15q11.2q13.1 (22,770,422_28,928,730)x3	6158 Kb	Paternal	15q11q13 duplication syndrome	Live birth with delayed speech and language development
6	21	14^+1^	11.642	dup (15q11.2-q13.1,5.43M)-M	Delayed growth and development for 1 week	Normal	arr [hg19] 15q11.2q13.1 (23,290,788_28,540,345)x3	5250 Kb	Maternal	15q11q13 duplication syndrome	Terminate of pregnancy
7	30	16	31.6	dup (15q11.2-q13.1,5.22M)	Normal	Normal	arr [GRCh37] 15q11.2q13.1 (23,277,436_28,644,578)x3	5367 Kb	Paternal	15q11q13 duplication syndrome	Terminate of pregnancy
8	34	17^+3^	21.796	dup (15q11.2-q13.1,5.43M)-M	Normal	46,XN,inv (9) (p12q13),dup (15) (q11.2q13.1)	arr [GRCh37] 15q11.2q13.1 (23,290,788_28,560,664)x3	5270 Kb	Maternal	15q11q13 duplication syndrome	Live birth and loss to follow up
9	24	23^+1^	34.835	dup (15q11.2-q13.1,5.43M)	Normal	dup (15) (q11.2q13)	arr [GRCh37] 15q11.2q13.1 (23,286,423_28,644,578)x3	5358 Kb	Unknown	15q11q13 duplication syndrome	Terminate of pregnancy
10	24	16^+1^	10.385	dup (15q11.2-q13.1,5.43M)-M	Normal	Normal	Normal	—	—	Normal	Normal
11	26	23^+1^	15.066	dup (15q13.1-q13.3,3.78M)-M	Choroid plexus cyst	—	—	—	—	—	Loss to follow up

### Karyotyping analysis of high-risk cases for 15q11q13 CNVs

Among the 4 cases identified as high-risk for 15q11q13 deletion, chromosomal karyotyping was not performed on 1 case, while the remaining 3 cases exhibited no karyotypic abnormalities. In the cohort of 7 cases identified as high-risk for duplication, chromosomal karyotyping confirmed 15q11q13 duplication in 2 cases, identified 1 case with 15q11q13 duplication in conjunction with chromosome 9 inversion, and revealed normal karyotypes in 3 cases; 1 case did not undergo amniocentesis.

### Amniocentesis for CMA analysis

Among the 4 cases with high-risk NIPS findings for the 15q11q13 deletion, CMA confirmed the deletion in 3 cases, with fragment sizes of approximately 5–6 Mb. The CMA results were concordant with the NIPS findings. The remaining case did not undergo CMA verification. In the cohort of 7 cases in which NIPS showed a high risk for a 15q11q13 duplication, CMA confirmed the duplication in 5 cases, identified normal findings in 1 case, and was not conducted in the remaining case (Figure 2). Based on the 9 cases that proceeded with invasive testing, CMA confirmed 8 true-positive results, yielding a PPV of 88.89% (8/9). Notably, among the 11 cases initially flagged as high-risk by NIPS, 2 declined amniocentesis and were excluded from this calculation. Therefore, the PPV may be overestimated if these untested cases are considered.

**FIGURE 2:**
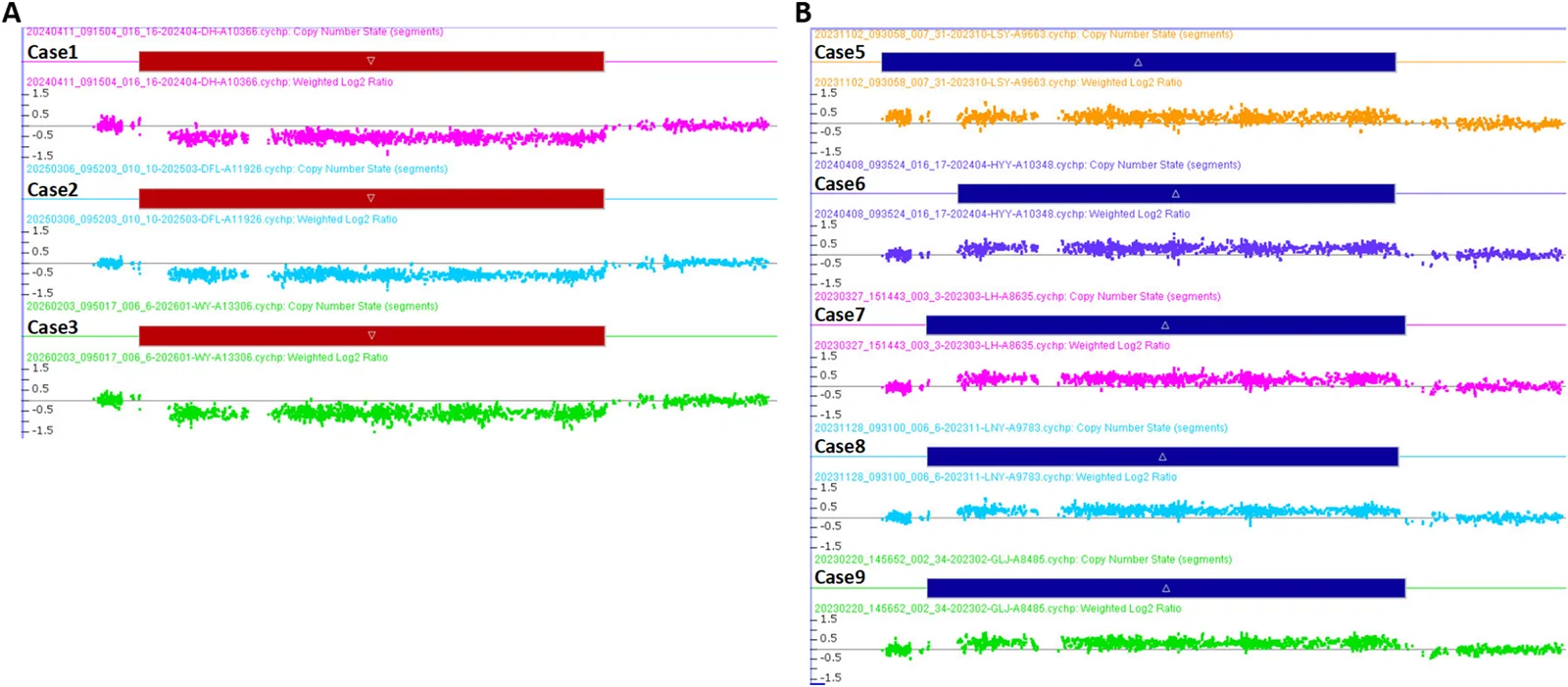
CMA results. **(A)** CMA identified deletion of 5.13 Mb, 5.11 Mb, and 5.13 Mb in the 15q11q13 region. **(B)** CMA identifies duplication of 6.01 Mb, 5.13 Mb, 5.24 Mb, 5.15 Mb, and 5.23 Mb in the 15q11q13 region.

### Ultrasound findings

Among the 8 true-positive cases, which included 3 instances of 15q11q13 deletion and 5 instances of 15q11q13 duplication, only 1 case of duplication was associated with growth and developmental delay. In contrast, the remaining 7 true-positive cases exhibited no significant ultrasound anomalies.

### Genetic origin and pregnancy outcomes

Among the 4 cases with 15q11q13 deletions, Cases 1 and 2 were confirmed by MS-MLPA as maternal inheritance, and both had termination of pregnancy after genetic counseling (Figures 3A,B). Case 3 chose to undergo termination without parental origin testing. Case 4 did not undergo prenatal diagnosis and was subsequently lost to follow-up.

**FIGURE 3:**
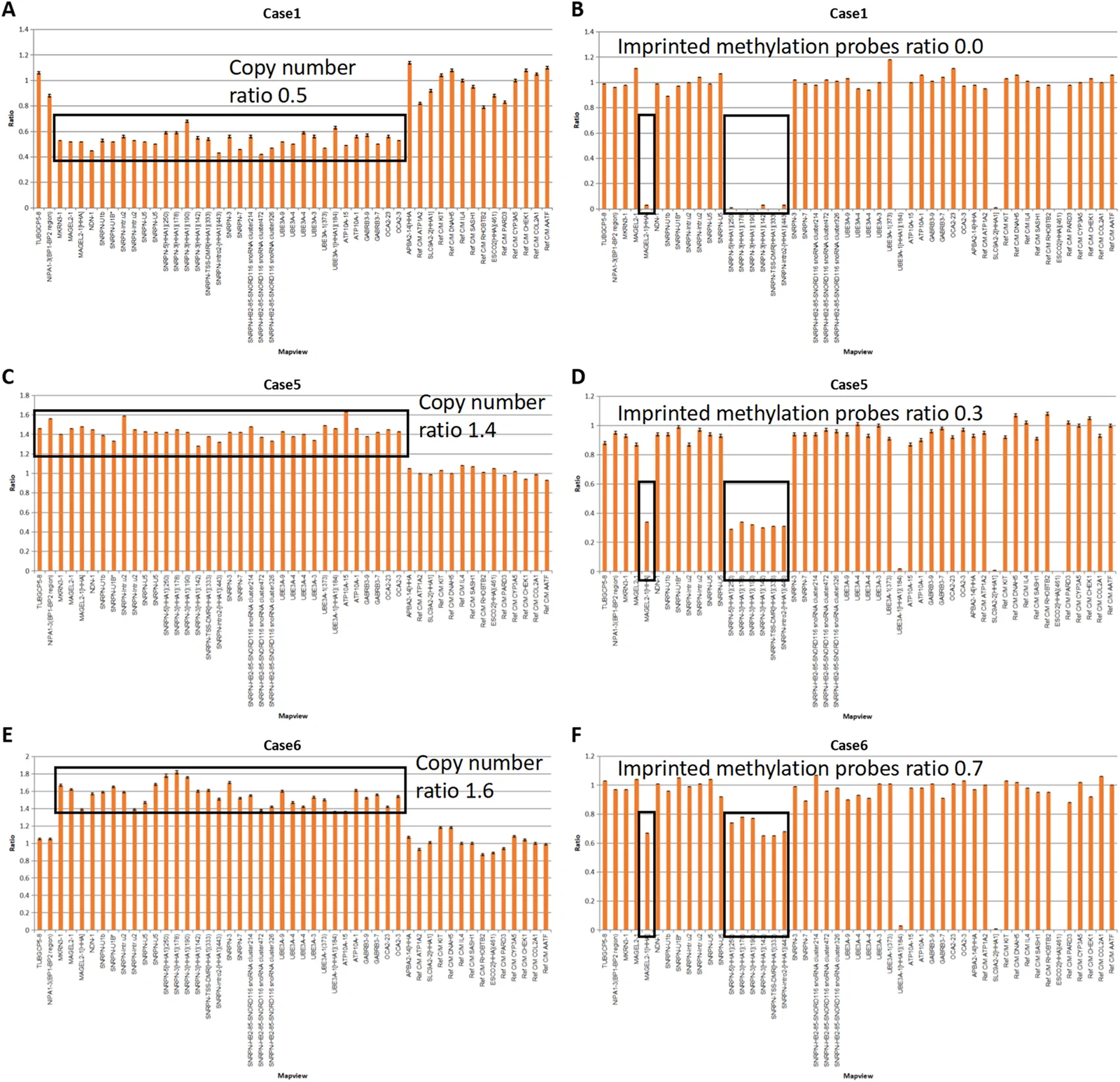
MS-MLPA results for the parental origin of 15q11q13 CNVs. **(A,B)** MS-MLPA analysis of Case 1. A deletion at the 15q11 region is shown in **(A)**, and maternal inheritance of the deletion is demonstrated in **(B)**. **(C,D)** MS-MLPA analysis of Case 5. A duplication at the 15q11 region is shown in **(C)**, and paternal inheritance of the duplication is demonstrated in **(D)**. **(E,F)** MS-MLPA analysis of Case 6. A duplication at the 15q11 region is shown in **(E)**, and maternal inheritance of the duplication is demonstrated in **(F)**.

Cases 5–9 were identified as carriers of 15q11q13 duplications. Parental origin testing by MS-MLPA was conducted for Cases 5 and 6, revealing paternal and maternal duplications, respectively (Figures 3C–F). In Case 5, a male infant was delivered by cesarean section at 36^+5^ weeks of gestation, with a birth weight of 2.95 kg and an Apgar score of 10; no abnormalities were detected at birth. At the 2-year follow-up, the child exhibited only a mild language delay compared with peers, while growth parameters, including height and weight, remained within normal ranges. In Case 6, the pregnant woman opted to terminate the pregnancy following comprehensive genetic counseling. CMA of the parental peripheral blood confirmed paternal and maternal duplications in cases 7 and 8, respectively. The woman in Case 7 opted for termination of pregnancy, whereas the woman in Case 8 decided to continue the pregnancy after receiving genetic counseling. At 40 weeks of gestation, the woman in Case 8 delivered a female infant by vaginal delivery, with a birth weight of 2.9 kg and an Apgar score of 10. No obvious abnormalities were observed at birth. However, at the 2-year follow-up, the parents declined further assessment.

## Discussion

This study evaluated the efficacy of NIPS for detecting CNVs in the 15q11q13 region in a large cohort of 90,693 pregnant women from southern China. With a PPV of 88.89% confirmed by CMA confirmation, we demonstrated that NIPS is a highly effective screening tool for this clinically significant genomic region. However, it is important to acknowledge that our study design precludes the calculation of sensitivity, specificity, negative predictive value, and false-negative rate, as invasive prenatal diagnosis and postnatal follow-up were offered exclusively to cases with high-risk NIPS results. Nonetheless, among the eight true-positive cases, the absence of abnormal ultrasound findings in all but one positive case underscores the limitations of conventional prenatal imaging for identifying neurodevelopmental disorders and reinforces the clinical utility of NIPS as a primary screening strategy.

In the 15q11q13 region, a cluster of imprinted genes is regulated by an imprinting center. Deletions on the maternal allele are associated with the development of AS, while deletions on the paternal allele are linked to PWS (Butler, 2026). The clinical effects of these parent-specific deletions are largely attributed to the differential expression of key genes in this cluster, particularly UBE3A. UBE3A encodes a ubiquitin protein ligase that is specifically expressed in the brain and is subject to maternal imprinting. Mutations, deletions, or imprinting defects in maternally inherited UBE3A can reduce its expression, leading to AS. In contrast, overexpression of maternally inherited UBE3A is correlated with an elevated risk of autism spectrum disorder and related conditions (Krzeski et al., 2024; Yang and Huang, 2025). The two cases of deletions identified in this study were confirmed to be maternally inherited, indicating that the fetuses were likely to develop clinical phenotypes associated with AS. Following comprehensive genetic counseling, both pregnant women chose to terminate their pregnancies.

Duplications of the imprinted 15q11q13 region cause Dup15q syndrome (Viamontes et al., 2022; Dias et al., 2024). Dup15q syndrome arises from two main mechanisms: a marker chromosome [idic (15)] that causes tetrasomy, and an interstitial duplication [int dup (15)] that causes trisomy. The phenotypic diversity in Dup15q syndrome is driven by differences in duplication fragment size, which are influenced by mutation type and breakpoint location. A parent-of-origin effect is evident in this genomic region, with maternal duplications conferring a heightened risk of adverse phenotypes (Bisba et al., 2024; Parijs et al., 2024). While paternal duplications are generally correlated with a normal phenotype, they have occasionally been reported in cases of autism (Urraca et al., 2013) and epilepsy (Marini et al., 2013). Overall, duplication of this region is primarily associated with neurodevelopmental disorders, including developmental delay, autism, epilepsy, and hypotonia (Bisba et al., 2024; Dias et al., 2024; Tamada and Takumi, 2025). In this study, 5 cases of 15q11q13 duplication were identified, of which 4 underwent parental origin testing by MS-MLPA or CMA. Among these, 1 case was identified as a paternal duplication and another as a maternal duplication. The infant with the paternal duplication displayed no significant abnormalities, aside from a mild language delay compared with peers. However, the parents of the infant with the maternal duplication declined further follow-up. The limited number of confirmed duplication cases with postnatal follow-up results in a scarcity of clinical phenotypic follow-up information, which represents a significant limitation of this study. Future work with larger cohorts will be essential to enhance our understanding of the prenatal and postnatal phenotypes of duplications in this region and to facilitate early diagnosis.

NIPS has demonstrated high sensitivity and specificity for screening trisomy 21, trisomy 18, and trisomy 13, and is now widely applied in clinical practice (Liu et al., 2023; Xiang et al., 2023; Wei et al., 2023). With increasing sequencing depth, advancements in bioinformatics analysis techniques, and the continuous accumulation of clinical data, the scope of NIPS has progressively expanded to include the detection of CNVs. The prevalence of pathogenic CNVs within the general pregnant population is estimated at approximately 1.6%–1.7% (Evans et al., 2016). Previous research has shown that NIPS can achieve a PPV exceeding 90% for specific genomic regions, such as the 22q11.2 region (Xiang et al., 2025; Soster et al., 2023). In this study, among 90,693 pregnant women who were screened, 11 cases were identified as high-risk for anomalies in the 15q11q13 region. Among these, 9 cases proceeded with prenatal diagnostic procedures, and 8 cases were confirmed as true positives by CMA, giving a positive predictive value of 88.89% (8/9). However, this PPV should be interpreted with caution. Two of the 11 high-risk cases declined amniocentesis, and their true statuses remain unknown. If these untested cases were assumed to be false positives, the PPV would decrease to 72.7% (8/11). These findings are consistent with a recent study by Peng et al., who reported 15q11q13 duplications in 3 of 69,605 pregnancies screened by NIPS in southern China, further supporting the utility of NIPS for detecting CNVs in this region across different cohorts (Peng et al., 2025). In our cohort, all positive cases were identified by NIPS screening. Notably, with the exception of 1 case of duplication associated with fetal growth restriction, no significant abnormal ultrasound findings were detected in the other positive cases during prenatal assessments. It is important to note that genomic disorders, such as Dup15q syndrome, may lack detectable sonographic features during the prenatal period. However, they are significantly associated with severe neurodevelopmental disorders and multisystem diseases. Consequently, NIPS facilitates early detection of high-risk fetuses during the first trimester and improves guidance for subsequent prenatal diagnostic procedures.

Among the cases identified as high-risk for duplications via NIPS, one instance was determined to be a false positive. In this case, NIPS suggested that the duplication was most likely attributable to maternal DNA interference; however, this possibility was not verified by CMA analysis of the pregnant woman. This finding highlights that, although NIPS has a relatively high positive predictive value for screening this region, false-positive results can still occur in a minority of cases. An analysis based on the principles of NIPS suggests that false positives may arise from: (1) confined placental mosaicism (CPM), in which chromosomal abnormalities are present only in the placenta, potentially affecting NIPS results because fetal DNA in maternal blood primarily originates from trophoblasts; (2) interference from maternal DNA, which may contain chromosomal abnormalities and skew the results; and (3) insufficient sequencing depth and bioinformatics limitations, which can reduce accuracy (Hartwig et al., 2017). Consequently, invasive prenatal diagnosis is essential following a high-risk NIPS result.

Our findings demonstrate that NIPS serves as a reliable primary screening method for detecting CNVs in the 15q11q13 region, particularly given that standard prenatal ultrasound frequently fails to identify structural abnormalities in most positive cases. This finding holds clinical significance, as Dup15q syndrome and AS are predominantly neurodevelopmental disorders that may not present with clear sonographic markers during gestation. By identifying high-risk fetuses in the first trimester, NIPS allows timely invasive diagnostic confirmation via amniocentesis, followed by CMA or MS-MLPA to determine the precise size, breakpoints, and parental origin of the variant—information that is critical for accurate prognosis and genetic counseling. Parental origin testing should be routinely integrated into the diagnostic workup, as maternal and paternal duplications carry substantially different neurodevelopmental risks, and maternal deletions are universally associated with AS. Before these findings can be widely applied, larger prospective studies with thorough postnatal follow-up are necessary to clarify genotype-phenotype correlations and develop evidence-based counseling and management guidelines.

Major strengths of this study include the large, unselected southern Chinese screening population (90,693 pregnancies), the use of CMA as a confirmatory diagnostic method for all invasive cases, and the inclusion of parental origin testing for a subset of cases. The PPV of 88.89% for this specific region provides robust real-world performance data that can directly inform clinical practice. However, several limitations must be acknowledged. The small number of duplication cases with postnatal follow-up (only one paternal case) severely limits our ability to draw definitive conclusions regarding phenotype-genotype correlations. The false-positive case lacked maternal CMA validation, precluding a complete understanding of its etiology. In addition, karyotype analysis confirmed only 2 of 8 CMA-positive cases, highlighting its limited resolution for this region, although it remains useful for detecting concurrent large structural rearrangements. The single-center design and the voluntary nature of prenatal diagnosis and follow-up may introduce selection biases that affect the generalizability of our findings.

## Conclusion

In conclusion, NIPS effectively screens for CNVs in the 15q11q13 region, with a positive predictive value of 88.89%, making it a valuable tool for early detection of syndromic disorders linked to neurodevelopmental impairment and unremarkable prenatal ultrasound findings. However, due to the lack of systematic follow-up in NIPS-negative cases, the sensitivity, specificity, negative predictive value, and false-negative rate of this approach remain to be determined in future prospective studies. As a screening tool, NIPS-positive results should be confirmed by invasive prenatal diagnostics like amniocentesis. Techniques such as CMA and MS-MLPA may be used to determine the size, location, and parental origin of CNVs, thereby facilitating genetic counseling and clinical decision-making.

## Data Availability

The original contributions presented in the study are publicly available. This data can be found in the GEO repository with the accession number GSE348526. For any reasonable inquiries or requests for further information, please contact the corresponding author.
